# Clinical Notes on Antiseptics

**Published:** 1886-06-20

**Authors:** A. Mitchell

**Affiliations:** Los Angeles


### CLINICAL NOTES ON ANTISEPTICS.

By A. Mitchell, M.D., Los Angeles.

Antiseptics have been much talked and written about during the last few years, and a great advance has been made in antiseptic surgery.

Having spent sometime in the principal hospitals of New York during the past two years, I found that corrosive sublimate was used as an antiseptic, to the almost total exclusion of other antiseptics that have been in use formerly, except for instruments, when the carbolic acid was used. In all cases where I have used it the result has been very satisfactory, and in many cases was surprised at the rapidity with which wounds would heal when this antiseptic was used.

Case: A boy, aged eight years, had about half of his fore-finger taken off in a straw-cutter. Without resecting the bone, in order to get skin to cover the stump, I dressed it with antiseptic sublimate gauze, and told him to return in eight or ten days. At the end of ten days the stump had granulated over very nicely, and there was not the least unhealthy odor about it.

In cases where intrauterine injections were called for, I have found that a 1 in 3,000 solution worked like a charm, quickly removing the fetid odor. In all such cases it is advisable to use a return tube for the escape of the fluid.

The sublimate solution makes an admirable wash in gonorrhœal and phlectenular conjunctivitis.

The strength used will vary in different cases from 1 in 500 to 1 in 10,000. One in 10,000 will destroy micrococci. One in 2,000 would be safe to use in washing out abscesses.

A convenient way to carry this solution, in order to have it always on hand, and, too, in regard to accuracy, is:

℞. Corrosive sublimate........................grs. xxx,
Soda chloride...............................grs. x,
Glycerin...................................℥ j.

M. One drachm of this to the pint of water will make a solution of 1 in 2,000, and more water can be added until we get the strength required.

To prepare sponges, whip them thoroughly, then soak in water for twenty-four hours; whip again and then soak in Labaraque's solution 1 part to 5 of water for five hours, then put through permanganate of potash solution, grs. 5 to water ℥ 1. Let sponges remain in this for forty minutes, then take out and rinse and put in oxalic acid solution 1 in 50 for 15 minutes, then rinse in clear water, when they will be ready to use in sublimate solution at any time.

For the gauze dressing, take cheese cloth and boil for two or three hours, and then put it through same process as sponges and soak in sublimate solution for a few hours; a little cochineal may be added to give reddish tint. It is said that when a dressing loses this tint that it has lost its antiseptic properties. A little iodoform dusted over the sublimate gauze makes the iodoform gauze. With a free use of drainage tubes, a fountain syringe and a sublimate solution 1 in 25,000 and the gauzes in all surgical operations are much more readily cared for than they formerly were.

Drainage tubes should reach well down to the bottom of the wound, and then thoroughly washed out as often as required to keep the discharge healthy. Chicken and turkey bones make much better drainage tubes than rubber—at least, such has been my experience.

As physicians we may differ in our opinions as regards micrococci, bacteria, and poison germs of whatever variety, and the value of antiseptic, yet I believe that we have faith in antiseptics just in accordance with which we have followed it in the minutiæ. —*California Practitioner.*

